# Developmental Exposure to a Toxic Spill Compromises Long-Term Reproductive Performance in a Wild, Long-Lived Bird: The White Stork (*Ciconia ciconia*)

**DOI:** 10.1371/journal.pone.0034716

**Published:** 2012-04-18

**Authors:** Raquel Baos, Roger Jovani, David Serrano, José L. Tella, Fernando Hiraldo

**Affiliations:** 1 Department of Conservation Biology, Estación Biológica de Doñana (CSIC), Isla de la Cartuja, Sevilla, Spain; 2 Department of Evolutionary Ecology, Estación Biológica de Doñana (CSIC), Isla de la Cartuja, Sevilla, Spain; University of Lethbridge, Canada

## Abstract

**Background/Objective:**

Exposure to environmental contaminants may result in reduced reproductive success and long-lasting population declines in vertebrates. Emerging data from laboratory studies on model species suggest that certain life-stages, such as development, should be of special concern. However, detailed investigations of long-term consequences of developmental exposure to environmental chemicals on breeding performance are currently lacking in wild populations of long-lived vertebrates. Here, we studied how the developmental exposure to a mine spill (Aznalcóllar, SW Spain, April 1998) may affect fitness under natural conditions in a long-lived bird, the White Stork (*Ciconia ciconia*).

**Methodology:**

The reproductive performance of individually-banded storks that were or not developmentally exposed to the spill (i.e. hatched before or after the spill) was compared when these individuals were simultaneously breeding during the seven years after the spill occurred (1999–2005).

**Principal Findings:**

Female storks developmentally exposed to the spill experienced a premature breeding senescence compared with their non-developmentally exposed counterparts, doing so after departing from an unusually higher productivity in their early reproductive life (non-developmentally exposed females: 0.5±0.33SE fledglings/year at 3-yr old *vs*. 1.38±0.31SE at 6–7 yr old; developmentally exposed females: 1.5±0.30SE fledglings/year at 3-yr old *vs*. 0.86±0.25SE at 6–7 yr old).

**Conclusions/Significance:**

Following life-history theory, we propose that costly sub-lethal effects reported in stork nestlings after low-level exposure to the spill-derived contaminants might play an important role in shaping this pattern of reproduction, with a clear potential impact on population dynamics. Overall, our study provides evidence that environmental disasters can have long-term, multigenerational consequences on wildlife, particularly when affecting developing individuals, and warns about the risk of widespread low-level contamination in realistic scenarios.

## Introduction

Long-term consequences of exposure to environmental contaminants upon individual performance and wildlife population dynamics have long been a focus of research interest for several disciplines including ecotoxicology, endocrinology (endocrine disruption) and population biology [1]–[6]. Developmental exposure to these chemicals, even at low levels, may be particularly detrimental, with potential long-term effects on reproduction and ultimately individual fitness [7]–[9]. To date, empirical support for these developmental effects has come almost entirely from laboratory studies on model vertebrate species [10]–[13], as establishing such relationships in the wild requires long-term, individual-based population surveys which have generally been prohibitive [2], [14]. Although laboratory experiments are essential for establishing possible effects on individuals exposed to specific contaminants, they usually do not represent biologically relevant exposure to ambient concentrations of complex mixtures of chemicals (but see [9]). In addition, the diversity of animals' life histories and of environmental stressors they usually have to deal with in nature, introduces the possibility of specific responses to contaminants under field conditions that may not be evident in controlled laboratory experiments [15], [16].

Far from the controlled experimental conditions in laboratories, toxic spills often constitute a good example of such complex mixtures of chemicals, whose exposure threaten both ecosystems and human health. After a spill, dead animals and devastated landscapes become front-page news, and adverse short-term consequences are often relatively rapid and easy to determine [17], [18]. However, once the media and general public lose interest, delayed direct and indirect long-term effects may also occur, being even more detrimental and difficult to document [1], [19]. Moreover, it is often the case that no previous studies have been conducted in the affected areas and on species of concern [20], still less that long-term monitoring of individuals has been performed. However, these data are essential to evaluating some long-term effects of the exposure to contaminants, especially of those occurring during critical life cycle stages, such as development, which may have profound impacts on fitness only detectable with longitudinal studies at individual level.

**Figure 1:**
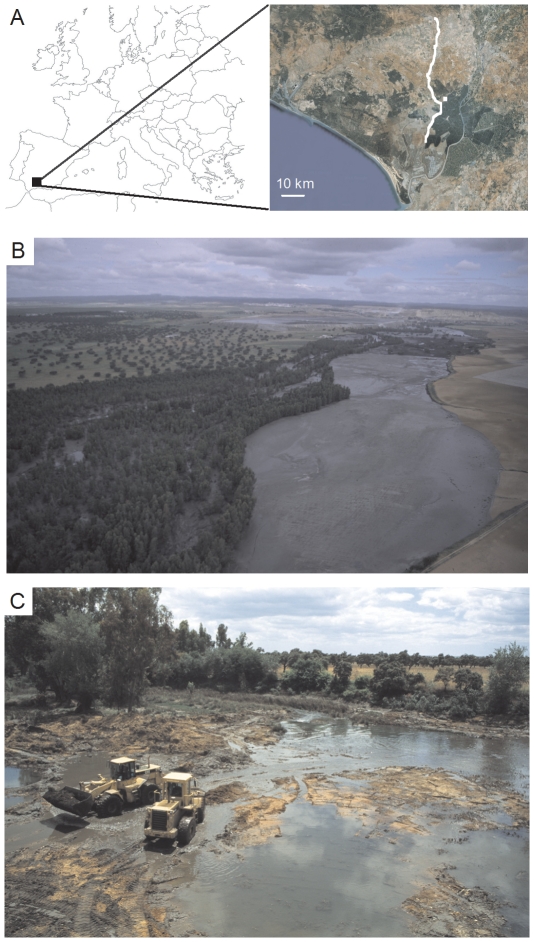
The Aznalcóllar mine accident struck one of Europe's most important wetland reserves. Geographical location of the study area. The area flooded by the mine spill in 1998 is drawn in white colour. The breeding location of white storks is also indicated (square) (**A**). Six million cubic meters of acidic waste ran down (from North to South) the Guadiamar River, leaving a toxic mud tongue 40 Km long and 400 m wide up to the Doñana World Heritage Site and International Biosphere Reserve (**B**). Clean-up operations extended for more than a year removing seven million cubic meters of mud and contaminated soils (**C**). Pictures from panels (***B***) and (***C***) are courtesy of Consejería de Medio Ambiente, Junta de Andalucía.

**Figure 2:**
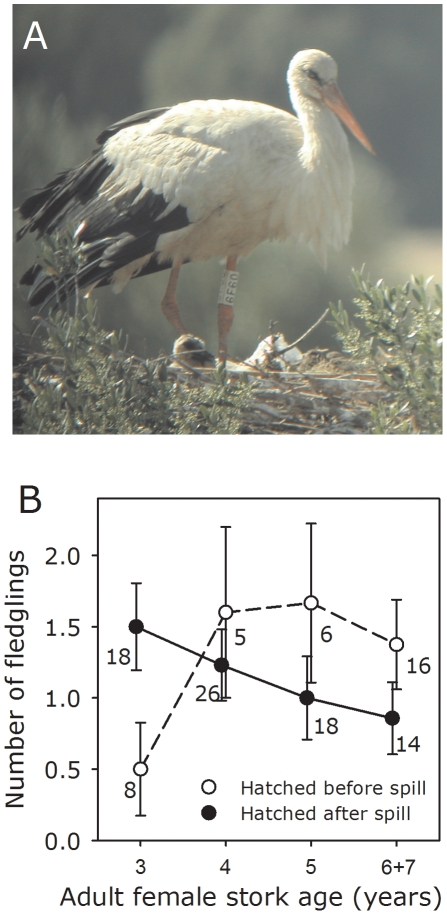
Breeding performance of female white storks nesting in the spill-affected area. Banded white stork (with unique plastic-coded leg band) breeding in the affected area (**A**). Number of fledglings raised by female storks hatched before (non-developmentally exposed) and after (developmentally exposed) the mine spill as a function of age (*B*). Bars represent the mean ± SE. Numbers correspond to individual breeding attempts. Picture by Roger Jovani.

In 1998, the dyke of the Aznalcóllar iron pyrite mine (SW Spain) (Fig. 1A) broke in one of the worst environmental disasters to occur worldwide [18]. Six million cubic meters of acidic waste ran down the Guadiamar River, leaving a toxic mud tongue 40 Km long and 400 m wide up to the Doñana World Heritage Site and International Biosphere Reserve (Fig. 1B). Clean-up operations started immediately (Fig. 1C), and a few years later the affected area was apparently recovered [21]. Nonetheless, short-term monitoring on humans and wildlife reported levels of metals and metalloids that, although often below threshold toxicity values, were higher in exposed than in reference populations [18], [21], [22]. Moreover, sublethal effects were found in exposed animals shortly after the accident, being especially conspicuous in the White Stork (*Ciconia ciconia*), a long-lived bird nesting in the area (Fig. 2A).

Monitoring on individual life histories of white storks was carried out for more than a decade in our study area (Fig. 1A) when the spill occurred (see Materials and Methods). Before the mine accident, nearly 2,000 nestling storks born in this area had been handled for ringing and biometric measures and no malformation was recorded. However, studies performed by the same research group for several years after the spill found that around 5% of the nestlings showed conspicuous malformations on bill and legs due to disrupted bone metabolism [23], [24]. Additionally, high levels of DNA damage were reported among storks born after the spill compared to reference ones [25]; cellular immune response was negatively associated to Cu concentrations [26]; and low Pb levels were found to enhance nestlings' adrenal stress response (i.e. induced production of corticosterone, the main glucocorticoid in birds) [27]. It has been reported that glucocorticoid production provoked by early-life environmental stress affects some cognition-based traits, some of which are directly related to fitness [28]. In fact, a lower probability of recruitment and long-term survival was recorded among nestling storks with higher adrenal stress response [29]. Concepts such as the special susceptibility of the developing organism to environmental stressors and early induction of latent effects are widely held within the scientific literature [30], [31], with an increasing recognition of the importance of developmental conditions (e.g. nutrient deficiency) on fitness parameters [32], [33]. However, as far as we know, the potential fitness consequences of the exposure to environmental chemicals during development remain completely unknown for wild, long-lived vertebrates.

Here, we provide what would be the first evidence of long-term fitness effects of low-level environmental contamination on developing individuals of long-lived species in the wild. Alternatively to the classical approach of comparing breeding parameters before and after a pollution event [34], [35], we simultaneously studied the breeding performance of individuals that were or not developmentally exposed to the spill (i.e. hatched before or after the spill) during a seven-year period after the spill occurred. Thus, we minimized potential confounding environmental effects associated with the spill (e.g. decreases in food availability and/or quality) other than the developmental exposure to contaminants.

## Materials and Methods

### Field procedures

Our study was conducted in the surroundings of the area directly flooded by the toxic waste in April 1998 (Fig. 1A), where there is one of the largest breeding aggregations of white storks known for both the Doñana marshlands and the breeding distribution of this species [36]. More than 200 pairs have been recorded breeding on top of wild olive trees per year, which have been intensively monitored since 1981. Annual monitoring included accurate nest counts, breeding outcome, and marking of most fledglings with plastic-coded leg bands easily readable at distance by using spotting scopes, to determine survival, recruitment, and dispersal of individuals. This long-term monitoring allowed knowing the identity and age of many adults breeding there in successive years. Further details of field protocols used in this study are presented elsewhere [29], [37].

To examine developmental spill effects on storks' breeding performance, we continued to band the fledglings hatched after the spill and collected individual-based data on reproduction during seven years following the mine accident (1999–2005). White storks are quite philopatric and show a high fidelity to their breeding site [38], [39], so we were able to record reproductive histories from several recruits to the study area.

We selected nests where the presence of a clutch was recorded. Storks were categorized as “non-developmentally exposed” to the spill if they had been hatched before 1998 and “developmentally exposed” if they had been hatched from 1998 onwards. We compared age-associated changes in breeding performance (clutch size and the number of fledglings produced, see [37] for details) between both groups of birds when breeding after the spill (see below). To avoid other potential factors affecting results (e.g. genetic factors, habitat characteristics, foraging areas) [34], [40], we focused exclusively on storks hatched in our study area before or after the spill and breeding in the same area following the mine accident (i.e. 1999–2005).

Breeders' age and sex were determined through banding records and molecular sexing [41] and/or observations of copulatory behaviour, respectively. The full data set consisted of 111 females and 138 males. None of them presented obvious deformities when banded as nestlings. We minimized disturbance to the study subjects by following protocols in concert with Spanish laws and prioritizing ethical considerations over scientific goals.

### Study design and statistical analyses

The fitness effects of contaminants on developing individuals can be confounded by other environmental effects such as a decrease in food availability and/or quality [42]. Although white storks have a surplus of food resources in the study area generated by the invasion and spread of what is currently their main prey, the North American red swamp crayfish (*Procambarus clarkii*) [43], we opted to compare the breeding performance of storks developmentally exposed or not to the spill just in the period after the spill. By this way, instead of classical approaches that compare pre- with post-spill breeding parameters, all our study subjects (developmentally exposed or not to the spill) were breeding under the same environmental conditions. Nonetheless, environmental aspects other than food availability and/or quality (e.g. rain episodes that might reduce breeding success [44]) could also vary between years during the post-spill period. Therefore, we included in our statistical analyses the year of breeding as a random factor, to minimize the potential, confounding effects of weather or any other unmeasured environmental (year) effects on reproduction. Bird identity and year of birth were also considered as random terms to control for unmeasured sources of between-individual and between-cohort variation. Since long-lived bird species are known to improve their breeding parameters with age [45], we also decided to control for age effects on breeding performance. After all, the mine accident offered us a “natural experiment” where the “treatment” was the exposure to contaminants during individual development, measuring breeding performance of birds during similar environmental conditions after the spill, while controlling for age and potential sources of variation (individual, cohort, and year of breeding) as random effects.

Generalized Linear Mixed Models (GLMMs) with Poisson error distribution and log link function were run using the GLIMMIX Procedure of SAS (SAS® v. 9.2). Clutch size and the number of fledglings were analyzed in relation to developmental exposure to the toxic spill (group factor with two levels: hatched before or after the spill, i.e. non-developmentally spill-exposed or developmentally spill-exposed, respectively) and age (either as linear or quadratic functions). Two-term interactions between the group factor and linear and quadratic functions of age (i.e. age×group and age^2^×group) were also included in the saturated model. Each explanatory variable was tested for statistical significance following standard backwards procedures, where non-significant (*P*>0.05) variables and interactions were sequentially removed from the saturated model. Bird identity, breeding year and year of birth were included as random terms to control for unmeasured sources of between individual, between-year, and between-cohort variations (see [46]). Moreover, a first-order autoregressive covariance structure (AR(1)) allowed the assessment of within-individual changes with age [47]. Females and males were analyzed separately to unravel potential sex-specific effects on reproduction and avoid pseudoreplication.

The Information Theory (IT) approach for model selection (where Akaike's Information Criterion or AIC is the most popular descriptor for selecting models) is increasingly considered superior to the classical Null Hypothesis Testing (NHT) approach and model simplification through stepwise regression (but see caveats of both approaches in [48]). We could not use AIC criteria here since the structure of our models, with random effects and non-normal distribution of errors (i.e. GLMMs), involves parameter estimation based on residual log pseudo-likelihood, while the AIC criteria use maximum likelihood, and there are warnings in the literature that AIC cannot be safely used in case of mixed models [49] (and references therein). Nonetheless, recent reanalyses of data [50] have shown that model simplification through stepwise regression and model selection based on IT yield similar results, suggesting there is not a method consistently better than others (see also [48]). Moreover, we provide our whole data set as Table S1 in the hope that further statistical developments will allow other researchers to reanalyze it under IT or other alternative approaches.

### Ethic statement

Storks returned to their nests just a few minutes after our inspection of nest content, rapidly resuming their normal breeding activities. Thus, our perturbation was supposed to be low. Moreover, we did a low number of visits each year, and we inspected nest contents from the ground with a mirror attached at the end of a long pole to minimize disturbance. The study carried out was conducted in agreement with the Environmental Agency of the Andalusian Government and the Spanish Ministry of Environment.

## Results and Discussion

In long-lived birds, age-related improvements in breeding performance are typically recorded during the early stages of the reproductive lifespan [45]. In storks, this predicted pattern was observed in females hatched before the spill. However, females hatched after the spill, and thus exposed during development, showed a striking decrease in breeding performance with age departing from a higher productivity than their non-developmentally exposed counterparts (Table 1, Fig. 2B). This is not only a population pattern driven by between-individual heterogeneity (e.g. selective appearance and disappearance of phenotypes) since even a stronger pattern was found in a longitudinal, within individual analysis of the females for which reproduction was monitored in multiple years [Repeated Measures, age×group (hatched before the spill): estimate ± SE = 0.896±0.284; *F*_1,30_ = 9.93, *P* = 0.004]. No effect, however, was detected in males (all *P*>0.153).

**Table 1:** Summary of results from the GLMM explaining the number of fledglings in female storks (n = 111 breeding attempts).

Effect	Estimate	SE	*F*-value (df)	*P*
intercept	1.044	0.505	2.07^a^ (5)	0.093
age	−0.222	0.117	0.32 (1,49)	0.574
group	−1.444^b^	0.863	2.80 (1,49)	0.101
age×group	0.351^b^	0.163	4.64 (1,49)	0.036
bird	0.117^c^	0.129		
year of birth	0.018^c^	0.076		
year of breeding	0.049^c^	0.078		

Number of fledglings was the response variable. Group (hatched before or after the spill) and female age were explanatory variables, and bird, year of birth (cohort) and year of breeding were treated as random effects. SE, standard error; df, degrees of freedom;

a *t*-Test;

b Estimate corresponds to the group of storks hatched before the spill (the level “hatched after the spill” is aliased);

c random effect. The estimated effects, SE, *F*-values and associated probabilities are shown for those variables that significantly improved the fit of the model (*χ^2^*/df = 1.06).

Studies monitoring populations over a period of time long enough for long-term spill impacts to be detected are scarce, especially when research requires pre-spill information [20]. Previous works conducted after oil tanker accidents reported spill-related effects on survival and short-term reproduction in wild vertebrates [1], [35], [51], [52]. However, to the best of our knowledge, this is the first study to link a spill exposure in early life to subsequent long-term effects on reproduction, ultimately affecting individual fitness, thus potentially linking animal life-history trade-offs with the developmental exposure to environmental contaminants. Moreover, our study design (see above) and data analysis allowed us to minimize the potential confounding effects of environmental variation on breeding parameters.

Over the past decades, a growing number of laboratory experiments on endocrine disruption suggest that exposure to low levels of contaminants at early stages in vertebrate development may impair reproduction later in life [7], [8], [10]–[12], [31]. Similarly, literature on humans and wild vertebrates has reported that adverse environmental conditions early in life (e.g. nutrient deficiency) may have long-term fitness costs [30], [53], but exposure to environmental contaminants has not been studied in this regard in a natural setting. Our results show that developmental exposure to environmental contaminants might affect long-term fitness, doing so in a complex and even in a, *a priori*, paradoxical manner, since we found a higher early fecundity followed by a premature reproductive senescence in the developmentally exposed females. The observational nature of our study does not allow us to completely discount the possibility that the decrease in productivity observed after the age of 4–5 years old may also be affected by the pollutants accumulation throughout adult life. However, we performed a complementary analysis to test whether the exposure time before the breeding attempt (number of years elapsed between the spill and each breeding attempt) affected females' productivity, showing no such effect (GLMM: *F_1_*_,50_ = 1.51, *P* = 0.224). This supports the role of developmental exposure in the pattern described above.

Life-history theory provides a potential explanation for that pattern. Since long-lived organisms have to make a series of decisions such as when to breed for the first time, how many times to reproduce, and how to trade-off limited resources between reproduction and maintenance or growth [54], life-history theory predicts individuals should increase reproductive investment when their residual reproductive value is low (“terminal investment” hypothesis) [55]–[57]. In previous studies, we showed impairments in both immune- and adrenal stress-response [26], [27], and disrupted bone metabolism [23], [24], and that spill exposure of nestling storks was linked to DNA damage even in birds hatched four years after the spill [25], [58]. We thus propose that these costly sub-lethal effects compromising health may act as physiological cues of reduced survival prospects and future reproduction. In this way, this lowering of residual reproductive value would lead storks to invest comparatively more in early reproduction. This may explain both early higher productivity and advanced breeding senescence in birds developmentally exposed to the toxic spill, due to the combined harmful effects of the spill-derived contaminants and to the early investment in reproduction challenging future reproduction [53], [59], [60]. The absence of similar results when using clutch size as the dependent variable (all *P*>0.208), suggests that egg composition and quality (e.g. antioxidants investment in eggs) [61] and/or parental effort (i.e. the amount of resources allocated to parental activities such as incubation, brooding, or feeding) [62] might be behind the observed pattern on the numbers of fledglings.

The compounds underlying such effects are difficult to determine because of the cocktail of contaminants in the mine spill [18]. Although metals and metalloids were the most abundant in the toxic sludge, organic compounds were also present [63], and all have been linked to adverse effects in humans and wildlife [3], [64]. Moreover, different contaminants may function differently in nature, and additive, synergistic or antagonistic effects are common, making it more difficult to isolate the effects of single compounds or subgroups of compounds on ecological traits in field studies. Although metals are very likely to constitute a major part of the pollutant stress in the stork population, and several studies supported their role in the sublethal effects found in spill-exposed nestling [23], [24], [26], [27], [58], we cannot exclude the possibility that contaminants other than those present in the spill (e.g. dioxin and related compounds, flame retardants, etc.) have parallel effects, even though the levels of some of these compounds in the area seem to be low [65]–[67].

With this study, we show that the exposure to environmental contaminants during development could contribute to age-specific changes in reproductive success in the same way as other stress factors [68], playing thus a role in determining life-history trajectories. Although we cannot identify the proximate basis for these changes, previous studies point out several non-exclusive mechanisms by which sub-lethal effects of the spill-derived contaminants may affect female storks' reproductive performance and rates of senescence. One possibility is given by the oxidative stress caused by reactive oxygen species (ROS). Evolutionary theories discuss it as a key process in senescence [68]. Oxidative stress in wildlife sentinel species other than birds has been reported to be linked to the Aznalcóllar mine spill [69], [70]. In storks, spill-exposed nestlings showed greater DNA damage in lymphocytes than reference birds [25], increasing with years [71], and being affected, in part, by the exposure to metals [58]. Being aware that associations between oxidative stress and life history traits are complex [72], it is difficult to determine whether the DNA damage measured in nestling storks is directly linked to the impaired later fecundity found here. However, a recent study using genetic analyses has reported that families of captive Zebra Finches (*Taeniopygia guttata*) with higher early resistance to oxidative stress (measured before the first breeding attempt) had more reproductive events during lifetime and delayed reproductive senescence [73]. Moreover, in Alpine Swifts (*Apus melba*), resistance to oxidative stress covaries with fecundity in females but not in males [74], supporting our sex-specific results. Several studies have revealed sex differences in response to stressful conditions in early life; females appear to be more sensitive to adverse developmental conditions when compared with males, exhibiting more evident long-term phenotypic effects [62] (and references therein). In addition, there is evidence that females are more vulnerable to the impairment of neural functions in response to Pb and other stressors [75]. Given the crucial role that hormones play in controlling (neuro)development, physiology and behaviour [4], [5], endocrine disruption might also be among the potential mechanisms involved in the effects we found here. Moreover, premature reproductive senescence has been experimentally reported in female rodents developmentally exposed to endocrine active substances [76].

Of special concern is that effects we report in this and previous studies were associated with low levels of environmental contaminants [26], [27], [58]. Low-dose exposure is particularly important because of its relevance to the levels experienced by the general human population. Several investigations have shown that some chemicals having adverse effects in wildlife are also detrimental for humans [5], [7], [13]. In fact, a recent study on children exposed to low Pb levels in early development has reported an impaired stress response similar to that previously reported in nestling storks also exposed to sub-lethal levels of this metal [77].

### Conclusions

Our study adds to a growing body of literature suggesting that toxic spills have the potential to affect wildlife for much longer periods than previously assumed (see e.g. [78]). Major environmental “disasters” are usually labelled as such because of their immediate and obvious impacts. However, our present findings provide empirical evidence that there are “hidden”, long-term fitness effects that also pose a threat to affected populations and ecosystems. We show that low-level environmental contamination may have long-term complex consequences on fitness of those individuals exposed during development that may not be evident in controlled laboratory experiments, and that may even lead to misleading interpretations. For instance, the higher fecundity of animals early in their reproductive life might diminish conservation concern, despite the fact, as we have shown here, that it may be the beginning of declining breeding performance, with a clear potential impact on population dynamics.

Although our study is based on observational data, and thus the causal relationships and mechanism(s) involved are difficult to identify, we believe that the suggested scenarios offer promising avenues for further investigations. Overall, our results encourage interdisciplinary approaches to these issues, demanding greater caution in short-term assessments of environmental impacts and supporting the long-claimed value of multigenerational studies using long-lived species to detect “hidden”, long-term effects of low-level environmental contamination. More generally, being aware that ambient exposure to low levels of chemicals in the form of mixtures is the rule rather than the exception [79], our study alerts that subtle harmful effects such as the ones found here may be more common among natural populations of vertebrates than has been appreciated to date. They may often go undetected or unrecognized in the absence of long-term monitoring plans, thus demanding greater efforts in this research area.

## Supporting Information

Table S1 **Data on reproductive parameters from white storks (** ***Ciconia ciconia*** **) breeding in the study area after the Aznalcóllar mine spill (1999–2005).** Bird code identifies individuals. Sex: 1 = males, 2 = females. Age in years. Group: 0 = hatched before the spill, 1 = hatched after the spill. * The exact number of eggs laid could be not determined.
(DOC)
