# Supplementary material for: Developmental Exposure to a Toxic Spill Compromises Long-Term Reproductive Performance in a Wild, Long-Lived Bird: The White Stork (Ciconia ciconia)
Source: PLoS One. 2012 Apr 18;7(4):e34716. doi: 10.1371/journal.pone.0034716 (PMC3329485; doi:10.1371/journal.pone.0034716)
Supplement: Table S1 — Data on reproductive parameters from white storks ( Ciconia ciconia ) breeding in the study area after the Aznalcóllar mine spill (1999–2005). Bird code identifies individuals. Sex: 1 = males, 2 = females. Age in years. Group: 0 = hatched before the spill, 1 = hatched after the spill. * The exact number of eggs laid could be not determined. (DOC) [file pone.0034716.s001.doc]

**Table S1. Data on reproductive parameters from White Storks (*Ciconia ciconia*) breeding in the study area after the Aznalcóllar mine spill (1999-2005).** Bird code identifies individuals. Sex: 1= males, 2= females. Age in years. Group: 0= hatched before the spill, 1= hatched after the spill. * The exact number of eggs laid could be not determined.

| **Bird code** | **Sex** | **Cohort** | **Year of**  **breeding** | **Age** | **Group** | **# eggs** | **# fledglings** |
| --- | --- | --- | --- | --- | --- | --- | --- |
| CC102 | 1 | 1992 | 1999 | 7 | 0 | * | 4 |
| CC146 | 1 | 1993 | 1999 | 6 | 0 | 2 | 0 |
| CC146 | 1 | 1993 | 2000 | 7 | 0 | 3 | 0 |
| CC147 | 1 | 1993 | 1999 | 6 | 0 | 4 | 3 |
| CC147 | 1 | 1993 | 2000 | 7 | 0 | 4 | 2 |
| CC153 | 1 | 1994 | 1999 | 5 | 0 | 4 | 2 |
| CC153 | 1 | 1994 | 2000 | 6 | 0 | * | 0 |
| CC153 | 1 | 1994 | 2001 | 7 | 0 | 3 | 3 |
| CC154 | 1 | 1994 | 2001 | 7 | 0 | * | 4 |
| CC157 | 1 | 1994 | 2001 | 7 | 0 | 5 | 2 |
| CC196 | 1 | 1994 | 1999 | 5 | 0 | 3 | 2 |
| CC196 | 1 | 1994 | 2000 | 6 | 0 | 4 | 0 |
| CC196 | 1 | 1994 | 2001 | 7 | 0 | 4 | 1 |
| CC227 | 1 | 1997 | 2003 | 6 | 0 | 4 | 3 |
| CC227 | 1 | 1997 | 2004 | 7 | 0 | 4 | 0 |
| CC290 | 1 | 1997 | 2000 | 3 | 0 | * | 3 |
| CC290 | 1 | 1997 | 2001 | 4 | 0 | 3 | 2 |
| CC290 | 1 | 1997 | 2002 | 5 | 0 | 3 | 2 |
| CC290 | 1 | 1997 | 2003 | 6 | 0 | 4 | 3 |
| CC290 | 1 | 1997 | 2004 | 7 | 0 | 4 | 2 |
| CC299 | 1 | 1997 | 2000 | 3 | 0 | 4 | 2 |
| CC299 | 1 | 1997 | 2001 | 4 | 0 | 4 | 4 |
| CC299 | 1 | 1997 | 2002 | 5 | 0 | 4 | 3 |
| CC300 | 1 | 1997 | 2002 | 5 | 0 | 4 | 0 |
| CC300 | 1 | 1997 | 2003 | 6 | 0 | 4 | 0 |
| CC301 | 1 | 1997 | 2002 | 5 | 0 | 3 | 0 |
| CC302 | 1 | 1997 | 2000 | 3 | 0 | 4 | 0 |
| CC302 | 1 | 1997 | 2001 | 4 | 0 | 4 | 3 |
| CC302 | 1 | 1997 | 2002 | 5 | 0 | 3 | 0 |
| CC302 | 1 | 1997 | 2004 | 7 | 0 | 4 | 3 |
| CC309 | 1 | 1997 | 2000 | 3 | 0 | * | 2 |
| CC309 | 1 | 1997 | 2001 | 4 | 0 | * | 1 |
| CC309 | 1 | 1997 | 2002 | 5 | 0 | 3 | * |
| CC309 | 1 | 1997 | 2004 | 7 | 0 | 4 | 2 |
| CC109 | 1 | 2000 | 2004 | 4 | 1 | 5 | 0 |
| CC109 | 1 | 2000 | 2005 | 5 | 1 | 4 | 0 |
| CC116 | 1 | 2000 | 2003 | 3 | 1 | 5 | 0 |
| CC116 | 1 | 2000 | 2004 | 4 | 1 | 3 | 2 |
| CC116 | 1 | 2001 | 2005 | 4 | 1 | 4 | 1 |
| CC119 | 1 | 2002 | 2005 | 3 | 1 | 4 | 1 |
| CC12 | 1 | 1998 | 2003 | 5 | 1 | 4 | 0 |
| CC12 | 1 | 1998 | 2004 | 6 | 1 | 4 | 2 |
| CC12 | 1 | 1998 | 2005 | 7 | 1 | 4 | 1 |
| CC120 | 1 | 2000 | 2003 | 3 | 1 | 4 | 0 |
| CC125 | 1 | 2000 | 2003 | 3 | 1 | 1 | 0 |
| CC127 | 1 | 2000 | 2004 | 4 | 1 | 3 | 2 |
| **Bird code** | **Sex** | **Cohort** | **Year of**  **breeding** | **Age** | **Group** | **# eggs** | **# fledglings** |
| CC13 | 1 | 1998 | 2002 | 4 | 1 | 3 | 0 |
| CC13 | 1 | 1998 | 2004 | 6 | 1 | 4 | 3 |
| CC13 | 1 | 1998 | 2005 | 7 | 1 | 3 | 0 |
| CC131 | 1 | 2000 | 2003 | 3 | 1 | 3 | 2 |
| CC131 | 1 | 2000 | 2004 | 4 | 1 | 3 | 1 |
| CC131 | 1 | 2000 | 2005 | 5 | 1 | 3 | 0 |
| CC132 | 1 | 2000 | 2005 | 5 | 1 | 4 | 0 |
| CC134 | 1 | 2000 | 2003 | 3 | 1 | 3 | 2 |
| CC134 | 1 | 2000 | 2004 | 4 | 1 | 4 | 0 |
| CC137 | 1 | 2002 | 2005 | 3 | 1 | 1 | 0 |
| CC14 | 1 | 1999 | 2002 | 3 | 1 | 4 | 2 |
| CC14 | 1 | 1999 | 2004 | 5 | 1 | 3 | 3 |
| CC159 | 1 | 2001 | 2004 | 3 | 1 | * | 1 |
| CC159 | 1 | 2001 | 2005 | 4 | 1 | 3 | 2 |
| CC16 | 1 | 1999 | 2002 | 3 | 1 | 4 | 1 |
| CC160 | 1 | 2001 | 2004 | 3 | 1 | 4 | 0 |
| CC160 | 1 | 2001 | 2005 | 4 | 1 | 4 | 0 |
| CC161 | 1 | 2001 | 2005 | 4 | 1 | 2 | 2 |
| CC162 | 1 | 2001 | 2004 | 3 | 1 | 5 | * |
| CC162 | 1 | 2001 | 2005 | 4 | 1 | 3 | 0 |
| CC163 | 1 | 2001 | 2004 | 3 | 1 | 3 | 3 |
| CC163 | 1 | 2001 | 2005 | 4 | 1 | 1 | 0 |
| CC168 | 1 | 2001 | 2004 | 3 | 1 | 4 | 2 |
| CC171 | 1 | 2001 | 2004 | 3 | 1 | 4 | 2 |
| CC171 | 1 | 2001 | 2005 | 4 | 1 | 5 | 0 |
| CC173 | 1 | 2002 | 2005 | 3 | 1 | 2 | 0 |
| CC175 | 1 | 2001 | 2004 | 3 | 1 | 1 | 1 |
| CC175 | 1 | 2001 | 2005 | 4 | 1 | 4 | 0 |
| CC180 | 1 | 2001 | 2005 | 4 | 1 | 4 | 0 |
| CC236 | 1 | 2002 | 2005 | 3 | 1 | 4 | 0 |
| CC237 | 1 | 2002 | 2005 | 3 | 1 | 2 | 1 |
| CC243 | 1 | 2002 | 2005 | 3 | 1 | 4 | 2 |
| CC246 | 1 | 2002 | 2005 | 3 | 1 | 3 | 0 |
| CC325 | 1 | 1998 | 2003 | 5 | 1 | * | 2 |
| CC325 | 1 | 1998 | 2004 | 6 | 1 | 2 | 2 |
| CC325 | 1 | 1998 | 2005 | 7 | 1 | 2 | 1 |
| CC326 | 1 | 1998 | 2001 | 3 | 1 | 3 | 2 |
| CC326 | 1 | 1998 | 2002 | 4 | 1 | 3 | * |
| CC326 | 1 | 1998 | 2004 | 6 | 1 | 4 | 2 |
| CC326 | 1 | 1998 | 2005 | 7 | 1 | 4 | 1 |
| CC33 | 1 | 1999 | 2002 | 3 | 1 | 4 | 2 |
| CC330 | 1 | 1998 | 2002 | 4 | 1 | 4 | 3 |
| CC330 | 1 | 1998 | 2004 | 6 | 1 | 5 | 3 |
| CC330 | 1 | 1998 | 2005 | 7 | 1 | 4 | 0 |
| CC331 | 1 | 1998 | 2004 | 6 | 1 | 4 | 2 |
| CC331 | 1 | 1998 | 2005 | 7 | 1 | 4 | 0 |
| CC334 | 1 | 1998 | 2004 | 6 | 1 | 3 | 2 |
| CC334 | 1 | 1998 | 2005 | 7 | 1 | 4 | 0 |
| CC336 | 1 | 1998 | 2001 | 3 | 1 | 4 | 2 |
| CC336 | 1 | 1998 | 2002 | 4 | 1 | 4 | 1 |
| CC336 | 1 | 1998 | 2003 | 5 | 1 | 1 | 0 |
| CC336 | 1 | 1998 | 2004 | 6 | 1 | 4 | 3 |
| CC336 | 1 | 1998 | 2005 | 7 | 1 | 5 | 0 |
| **Bird code** | **Sex** | **Cohort** | **Year of**  **breeding** | **Age** | **Group** | **# eggs** | **# fledglings** |
| CC337 | 1 | 1998 | 2004 | 6 | 1 | 5 | 2 |
| CC338 | 1 | 1998 | 2002 | 4 | 1 | * | 0 |
| CC338 | 1 | 1998 | 2003 | 5 | 1 | 4 | 4 |
| CC339 | 1 | 1998 | 2002 | 4 | 1 | 3 | * |
| CC339 | 1 | 1998 | 2003 | 5 | 1 | * | 2 |
| CC36 | 1 | 1999 | 2004 | 5 | 1 | 4 | 2 |
| CC36 | 1 | 1999 | 2005 | 6 | 1 | 4 | 2 |
| CC37 | 1 | 1999 | 2004 | 5 | 1 | 3 | 2 |
| CC37 | 1 | 1999 | 2005 | 6 | 1 | 4 | 2 |
| CC38 | 1 | 1999 | 2003 | 4 | 1 | 4 | 0 |
| CC41 | 1 | 1999 | 2002 | 3 | 1 | 4 | 0 |
| CC42 | 1 | 1999 | 2002 | 3 | 1 | 4 | 2 |
| CC42 | 1 | 1999 | 2005 | 6 | 1 | 2 | 0 |
| CC44 | 1 | 1999 | 2003 | 4 | 1 | * | 3 |
| CC44 | 1 | 1999 | 2004 | 5 | 1 | 3 | * |
| CC44 | 1 | 1999 | 2005 | 6 | 1 | 4 | 3 |
| CC46 | 1 | 1999 | 2003 | 4 | 1 | 5 | 3 |
| CC46 | 1 | 1999 | 2004 | 5 | 1 | 4 | 2 |
| CC46 | 1 | 1999 | 2005 | 6 | 1 | 4 | 2 |
| CC48 | 1 | 1999 | 2003 | 4 | 1 | 4 | 2 |
| CC48 | 1 | 1999 | 2004 | 5 | 1 | 3 | 2 |
| CC48 | 1 | 1999 | 2005 | 6 | 1 | 4 | 0 |
| CC49 | 1 | 1999 | 2002 | 3 | 1 | 4 | 2 |
| CC49 | 1 | 1999 | 2003 | 4 | 1 | 4 | 0 |
| CC49 | 1 | 1999 | 2005 | 6 | 1 | 4 | 3 |
| CC58 | 1 | 1999 | 2002 | 3 | 1 | 1 | 1 |
| CC58 | 1 | 1999 | 2003 | 4 | 1 | 4 | 3 |
| CC58 | 1 | 1999 | 2004 | 5 | 1 | 3 | 2 |
| CC58 | 1 | 1999 | 2005 | 6 | 1 | 4 | 2 |
| CC59 | 1 | 1999 | 2004 | 5 | 1 | 2 | 0 |
| CC6 | 1 | 1998 | 2002 | 4 | 1 | 4 | 1 |
| CC6 | 1 | 1998 | 2004 | 6 | 1 | 4 | 2 |
| CC6 | 1 | 1998 | 2005 | 7 | 1 | 4 | 2 |
| CC60 | 1 | 1999 | 2002 | 3 | 1 | 4 | * |
| CC61 | 1 | 1999 | 2002 | 3 | 1 | 3 | 0 |
| CC65 | 1 | 1999 | 2002 | 3 | 1 | 4 | * |
| CC66 | 1 | 1999 | 2003 | 4 | 1 | 4 | 3 |
| CC9 | 1 | 1998 | 2001 | 3 | 1 | 5 | 3 |
| CC92 | 1 | 2000 | 2003 | 3 | 1 | 2 | 0 |
| CC94 | 1 | 2000 | 2003 | 3 | 1 | 3 | 3 |
| CC94 | 1 | 2000 | 2004 | 4 | 1 | 4 | * |
| CC94 | 1 | 2000 | 2005 | 5 | 1 | 5 | 0 |
| CC96 | 1 | 2000 | 2004 | 4 | 1 | 4 | 2 |
| CC96 | 1 | 2000 | 2005 | 5 | 1 | 4 | 2 |
| CC98 | 1 | 2000 | 2003 | 3 | 1 | 4 | 2 |
| CC98 | 1 | 2000 | 2004 | 4 | 1 | 4 | 3 |
| CC98 | 1 | 2000 | 2005 | 5 | 1 | 4 | 1 |
| CC104 | 2 | 1992 | 1999 | 7 | 0 | 4 | 2 |
| CC145 | 2 | 1992 | 1999 | 7 | 0 | 3 | 2 |
| CC148 | 2 | 1993 | 1999 | 6 | 0 | * | 2 |
| CC148 | 2 | 1993 | 2000 | 7 | 0 | 4 | 1 |
| CC229 | 2 | 1997 | 2000 | 3 | 0 | 2 | 0 |
| CC231 | 2 | 1997 | 2000 | 3 | 0 | 4 | 0 |
| **Bird code** | **Sex** | **Cohort** | **Year of**  **breeding** | **Age** | **Group** | **# eggs** | **# fledglings** |
| CC283 | 2 | 1997 | 2000 | 3 | 0 | 2 | 2 |
| CC289 | 2 | 1997 | 2000 | 3 | 0 | 3 | 0 |
| CC294 | 2 | 1997 | 2000 | 3 | 0 | 3 | 2 |
| CC305 | 2 | 1997 | 2000 | 3 | 0 | 3 | 0 |
| CC308 | 2 | 1997 | 2000 | 3 | 0 | 3 | 0 |
| CC310 | 2 | 1997 | 2000 | 3 | 0 | 4 | 0 |
| CC212 | 2 | 1994 | 2001 | 7 | 0 | 3 | 3 |
| CC283 | 2 | 1997 | 2001 | 4 | 0 | * | 1 |
| CC289 | 2 | 1997 | 2001 | 4 | 0 | 4 | 3 |
| CC294 | 2 | 1997 | 2001 | 4 | 0 | 4 | 1 |
| CC308 | 2 | 1997 | 2001 | 4 | 0 | * | 3 |
| CC310 | 2 | 1997 | 2001 | 4 | 0 | 4 | 0 |
| CC229 | 2 | 1997 | 2002 | 5 | 0 | 5 | 0 |
| CC231 | 2 | 1997 | 2002 | 5 | 0 | 4 | 3 |
| CC283 | 2 | 1997 | 2002 | 5 | 0 | 3 | 2 |
| CC289 | 2 | 1997 | 2002 | 5 | 0 | 3 | 0 |
| CC308 | 2 | 1997 | 2002 | 5 | 0 | 3 | 2 |
| CC310 | 2 | 1997 | 2002 | 5 | 0 | 4 | 3 |
| CC229 | 2 | 1997 | 2003 | 6 | 0 | 1 | 0 |
| CC231 | 2 | 1997 | 2003 | 6 | 0 | 2 | 0 |
| CC305 | 2 | 1997 | 2003 | 6 | 0 | 2 | 1 |
| CC306 | 2 | 1997 | 2003 | 6 | 0 | 4 | 3 |
| CC308 | 2 | 1997 | 2003 | 6 | 0 | 4 | 3 |
| CC310 | 2 | 1997 | 2003 | 6 | 0 | 4 | 0 |
| CC229 | 2 | 1997 | 2004 | 7 | 0 | 1 | 0 |
| CC289 | 2 | 1997 | 2004 | 7 | 0 | 4 | 3 |
| CC305 | 2 | 1997 | 2004 | 7 | 0 | 3 | 2 |
| CC306 | 2 | 1997 | 2004 | 7 | 0 | 4 | 0 |
| CC308 | 2 | 1997 | 2004 | 7 | 0 | 2 | 0 |
| CC320 | 2 | 1998 | 2001 | 3 | 1 | 4 | 1 |
| CC321 | 2 | 1998 | 2001 | 3 | 1 | 5 | 2 |
| CC7 | 2 | 1998 | 2001 | 3 | 1 | 4 | 4 |
| CC10 | 2 | 1998 | 2002 | 4 | 1 | 5 | 0 |
| CC320 | 2 | 1998 | 2002 | 4 | 1 | 4 | 0 |
| CC321 | 2 | 1998 | 2002 | 4 | 1 | * | 0 |
| CC329 | 2 | 1998 | 2002 | 4 | 1 | 4 | 0 |
| CC47 | 2 | 1999 | 2002 | 3 | 1 | 2 | 1 |
| CC52 | 2 | 1999 | 2002 | 3 | 1 | 4 | 2 |
| CC53 | 2 | 1999 | 2002 | 3 | 1 | 1 | 0 |
| CC56 | 2 | 1999 | 2002 | 3 | 1 | 1 | 1 |
| CC57 | 2 | 1999 | 2002 | 3 | 1 | 4 | * |
| CC64 | 2 | 1999 | 2002 | 3 | 1 | 4 | 2 |
| CC68 | 2 | 1999 | 2002 | 3 | 1 | * | 3 |
| CC8 | 2 | 1998 | 2002 | 4 | 1 | 3 | 0 |
| CC110 | 2 | 2000 | 2003 | 3 | 1 | 2 | 2 |
| CC112 | 2 | 2000 | 2003 | 3 | 1 | 4 | 0 |
| CC123 | 2 | 2000 | 2003 | 3 | 1 | 4 | 0 |
| CC133 | 2 | 2000 | 2003 | 3 | 1 | 4 | 2 |
| CC135 | 2 | 2000 | 2003 | 3 | 1 | 3 | 0 |
| CC15 | 2 | 1999 | 2003 | 4 | 1 | 4 | 3 |
| CC321 | 2 | 1998 | 2003 | 5 | 1 | 3 | 0 |
| CC323 | 2 | 1998 | 2003 | 5 | 1 | 3 | 0 |
| CC333 | 2 | 1998 | 2003 | 5 | 1 | 3 | 0 |
| **Bird code** | **Sex** | **Cohort** | **Year of**  **breeding** | **Age** | **Group** | **# eggs** | **# fledglings** |
| CC34 | 2 | 1999 | 2003 | 4 | 1 | 4 | 2 |
| CC39 | 2 | 1999 | 2003 | 4 | 1 | 3 | 2 |
| CC47 | 2 | 1999 | 2003 | 4 | 1 | 3 | 3 |
| CC50 | 2 | 1999 | 2003 | 4 | 1 | 5 | 3 |
| CC56 | 2 | 1999 | 2003 | 4 | 1 | 4 | 3 |
| CC62 | 2 | 1999 | 2003 | 4 | 1 | 4 | 3 |
| CC64 | 2 | 1999 | 2003 | 4 | 1 | 4 | 0 |
| CC121 | 2 | 2000 | 2004 | 4 | 1 | 4 | 2 |
| CC124 | 2 | 2000 | 2004 | 4 | 1 | 3 | 1 |
| CC126 | 2 | 2000 | 2004 | 4 | 1 | 4 | 2 |
| CC128 | 2 | 2000 | 2004 | 4 | 1 | 5 | 0 |
| CC130 | 2 | 2000 | 2004 | 4 | 1 | 4 | 0 |
| CC133 | 2 | 2000 | 2004 | 4 | 1 | 4 | 0 |
| CC15 | 2 | 1999 | 2004 | 5 | 1 | 3 | 2 |
| CC170 | 2 | 2001 | 2004 | 3 | 1 | 4 | * |
| CC174 | 2 | 2001 | 2004 | 3 | 1 | 4 | 4 |
| CC179 | 2 | 2001 | 2004 | 3 | 1 | 4 | 2 |
| CC319 | 2 | 1998 | 2004 | 6 | 1 | 4 | 2 |
| CC328 | 2 | 1998 | 2004 | 6 | 1 | 4 | 2 |
| CC333 | 2 | 1998 | 2004 | 6 | 1 | 4 | 0 |
| CC39 | 2 | 1999 | 2004 | 5 | 1 | 3 | 0 |
| CC43 | 2 | 1999 | 2004 | 5 | 1 | 3 | 2 |
| CC47 | 2 | 1999 | 2004 | 5 | 1 | 4 | * |
| CC50 | 2 | 1999 | 2004 | 5 | 1 | 4 | 2 |
| CC56 | 2 | 1999 | 2004 | 5 | 1 | 5 | 3 |
| CC62 | 2 | 1999 | 2004 | 5 | 1 | 4 | 3 |
| CC64 | 2 | 1999 | 2004 | 5 | 1 | 4 | 0 |
| CC68 | 2 | 1999 | 2004 | 5 | 1 | 4 | 0 |
| CC121 | 2 | 2000 | 2005 | 5 | 1 | 4 | 2 |
| CC124 | 2 | 2000 | 2005 | 5 | 1 | 3 | 0 |
| CC126 | 2 | 2000 | 2005 | 5 | 1 | 4 | 0 |
| CC128 | 2 | 2000 | 2005 | 5 | 1 | 4 | 0 |
| CC130 | 2 | 2000 | 2005 | 5 | 1 | 3 | 1 |
| CC133 | 2 | 2000 | 2005 | 5 | 1 | 4 | 0 |
| CC139 | 2 | 2002 | 2005 | 3 | 1 | 2 | 0 |
| CC15 | 2 | 1999 | 2005 | 6 | 1 | 4 | 2 |
| CC158 | 2 | 2001 | 2005 | 4 | 1 | 4 | 0 |
| CC166 | 2 | 2001 | 2005 | 4 | 1 | 4 | 0 |
| CC170 | 2 | 2001 | 2005 | 4 | 1 | 4 | 2 |
| CC172 | 2 | 2001 | 2005 | 4 | 1 | 2 | 1 |
| CC174 | 2 | 2001 | 2005 | 4 | 1 | 4 | 2 |
| CC179 | 2 | 2001 | 2005 | 4 | 1 | 5 | 0 |
| CC186 | 2 | 2001 | 2005 | 4 | 1 | 4 | 3 |
| CC251 | 2 | 2002 | 2005 | 3 | 1 | 4 | 1 |
| CC319 | 2 | 1998 | 2005 | 7 | 1 | 4 | 2 |
| CC333 | 2 | 1998 | 2005 | 7 | 1 | 4 | 0 |
| CC34 | 2 | 1999 | 2005 | 6 | 1 | 3 | 0 |
| CC39 | 2 | 1999 | 2005 | 6 | 1 | 4 | 1 |
| CC43 | 2 | 1999 | 2005 | 6 | 1 | 4 | 0 |
| CC47 | 2 | 1999 | 2005 | 6 | 1 | 5 | 0 |
| CC50 | 2 | 1999 | 2005 | 6 | 1 | 4 | 2 |
| CC56 | 2 | 1999 | 2005 | 6 | 1 | 4 | 0 |
| CC62 | 2 | 1999 | 2005 | 6 | 1 | 4 | 1 |
| **Bird code** | **Sex** | **Cohort** | **Year of**  **breeding** | **Age** | **Group** | **# eggs** | **# fledglings** |
| CC68 | 2 | 1999 | 2005 | 6 | 1 | 4 | 0 |
| CC91 | 2 | 2000 | 2005 | 5 | 1 | 4 | 3 |
